# Effects of cell phone presence on the control of visual attention during the Navon task

**DOI:** 10.1186/s40359-023-01381-2

**Published:** 2023-10-12

**Authors:** Wenjuan Liu, Tomoya Kawashima, Kazumitsu Shinohara

**Affiliations:** 1 Department of Psychological Health, Wuhan Hankou Hospital, No. 7, 27 Side Road, Jiangan District, Wuhan City, 430010 Hubei Province China; 2 Department of Psychological Science, College of Informatics and Human Communication, Kanazawa Institute of Technology, Nonoichi, Japan; 3 Graduate School of Human Sciences, Osaka University, Osaka, Japan

**Keywords:** Cell phone presence, Attentional control, Navon task, Visual attention, Smartphone dependency

## Abstract

**Background:**

Although cell phones can provide great convenience to our lives, research has shown that they can also affect our behavior, even when not in use. It seems that having a cell phone nearby may not be ideal when the user needs to concentrate on work. However, little is known about whether cell phone presence specifically impairs attentional control.

**Methods:**

This study investigated whether cell phone presence can influence attentional control in the Navon task, which involves spatial switching of attention between global and local levels.

**Results:**

It was found that the reaction time for all types of trials decreased when the participants had a cell phone nearby compared to when they had a mobile battery nearby. It was also found that phone dependency led to more incorrect responses among participants, but this effect was independent of the influence of phone presence on the Navon task performance.

**Conclusions:**

These findings indicate that cell phone presence may have a positive influence on the perceptual process of the Navon letter, suggesting that the effects of phone presence are not always negative. One implication provided by this study is that it is possible to challenge the assertion that cell phones should always be excluded from the workplace by highlighting the positive effects of their presence.

## Introduction

Cell phones, which allow us to access a global repository of information, are effortlessly reached at our fingertips. Nevertheless, the general consensus is that excessive use of these devices is detrimental to our cognition [1–3]. Furthermore, there is growing evidence that exposure to environments where cell phones are present (even when not in use) can be detrimental to cognitive activities, such as visual search [4], cognitive capacity [5], and memory [6–8], thereby directly impairing the outcomes of cognitive activities [9–11].

In the cognitive psychology literature, the notion of attentional control, or the ability to efficiently allocate attentional resources to cognitive processes to achieve mental goals, is considered a crucial factor in successful cognitive activity [12–15]. It is plausible that the presence of a cell phone could impair cognitive abilities by affecting attentional control; however, the direct relationship between the two is yet to be fully explored.

### The potential link between cell phone presence and attentional control

In the theoretical framework of information processing, perceived information is processed in a conceptualized system known as working memory. According to the multicomponent model of working memory proposed by Baddeley [12], working memory consists of separate system elements for visuospatial information and for phonological information as well as a central executive managing their overall operation. Namely, the ability to control attention was typically considered one executive function in Baddeley’s model. On the other hand, Miyake et al. [15] examined the executive functions and identified three functions: shifting (i.e., switching between different tasks), inhibition (i.e., suppressing the inappropriate responses) and updating (i.e., managing and manipulating information temporarily stored in working memory). Attentional control, the ability to direct an individual’s attention to these executive processes in order to reach mental goals, are closely related to the function of this central executive system [14, 16]. Additionally, working memory capacity has often been used as an indicator of the efficiency of working memory function [17, 18]. Working memory capacity is defined as the amount of information that can be processed while it is temporarily stored in working memory. Individuals with high working memory capacity have been found to be better at attention tasks, such as the anti-saccade task [19], the Stroop task [20], and the dichotic-listening task [21] than individuals with low working memory capacity. Neuropsychological studies have also shown a greater level of activation in attentional control systems, such as dorsolateral prefrontal cortex and anterior cingulate cortex, during working memory capacity tasks [22], suggesting that working memory capacity is closely related to the efficiency of attentional control [23, 24].

However, evidence has shown that a high working memory capacity does not necessarily result in improved control of attention for shifting between different visual features at the perceptual stage. Goodhew [25] found that high working memory capacity individuals performed worse in the Navon task as compared to low working memory capacity individuals. The Navon task [26] is a task that requires attentional control for complex visual stimuli. In the Navon task, a single alphabet letter (global level) is composed of an array of small alphabet letters (local level), and the participant is asked to determine whether a specified letter is included at the global or local level. It is necessary to switch attention between global and local levels, and task performance is thought to depend on the ability to efficiently switch attention which is reflected by the working memory capacity. In this study, individuals performed a Navon task that required them to occasionally change their attentional scope. In one block of the Navon task, the target stimuli occurred at a certain hierarchical level (e.g., global) in 80% of the trials (majority trials), whereas in the remaining few cases (20%), the target stimuli occurred at the other hierarchical level (e.g., local, minority trials). The result showed that high working memory capacity individuals experienced a greater attentional shift cost (i.e., more time required to respond, or more incorrect response) than low working memory capacity individuals when the attentional shifts were needed (i.e., the hierarchy of the target changed from global to local). Goodhew argued that high working memory capacity means that more resources are available for full in-depth control attention that is optimal for the majority trials, correspondingly, their response to the minority trials was harmed by the facilitating effect on the majority trials. However, when working memory capacity is low, the lack of attentional resources results in inadequate attentional control in both majority and minority trials. Because the cost of attentional shift is more pronounced when there is a difference in the availability of attention between the majority trials and the minority trials, the cost of the attentional shift is not clearly observed in individuals with low working memory capacity. Thus, the working memory capacity may not be able to fully predict the efficiency of attention control when switching attention between different parts of the stimulus.

While high working memory capacity does not necessarily guarantee better attentional control, it is generally accepted that working memory capacity predicts one’s overall ability to control attention during information processing [23]. Consequently, studies [6, 7] concerning the effects of cell phone presence have also primarily focused on elucidating its impact on working memory capacity by initially considering attentional control as a unit. One previous study [6] reported that the cell phone presence can decrease performance on the operation span task (OSPAN task) [27] which is commonly used to measure working memory capacity. The OSPAN task combines mathematical problem-solving with the need to update and remember a sequence of randomly generated letters. Participants are required to solve mathematical problems while simultaneously keeping track of the changing sequence of letters. In this study, participants were randomly assigned to three different experimental conditions (i.e., phone-on-desk condition, phone-in-bag condition, and phone-absent condition). Participants with their phone nearby had significantly lower OSPAN scores than those in the phone-absent condition. The decreased performance on OSPAN task can be interpreted as an indication of fewer available attentional resources that can be used to coordinate the negative influence of the conflict between the cognitive processes of computation and memory. In support of this view, Ward and colleagues [6] suggested that the cell phone presence consumes attentional resources required for task performance, which may impair attentional control.

Previous studies have shown that the cell phone presence can affect attentional control. However, there has been no direct investigation of the impact of the cell phone presence on the shifting of visual attention. It is necessary to examine the impact on visual attention in more detail to clarify the effect of cell phone presence.

### Our objective in this study

This study aimed to investigate whether the cell phone presence can affect the ability to control attentional shifts. The presence of a cell phone automatically captures attentional resources. Since the amount of available attentional resources is limited, the attention drawn by the cell phone reduces the attentional resources available for use in working memory. This is akin to a situation where working memory is mentally taxed and available attentional resources are in short supply. When working memory is under mental strain, top-down attentional control is adversely affected [28], resulting in a less stable executive function control [29]. Therefore, we hypothesized that it would become more challenging to sustain attention on a single object, making attentional shifts more likely to occur. Our hypothesis was that the cell phone presence can facilitate the ability to shift visual attention which was assessed by using the Navon task. In the current Navon task, the mental goals (i.e., to determine the stimulus at a certain level) of the two consecutive trials remain the same in most cases, but occasionally change (e.g., the mental goal of the n-1 trial is to determine the stimulus at the global level, while the mental goal of the n trial is to the stimulus at the local level). The frequency of target-level switching was 25% (20% in [25]) and the occurrences of minor switching trials were pseudorandomized (see Method). Accordingly, we predicted that participants would respond faster and more accurately to the trials in which the target-level is varied from the prior trial when in an environment with a cell phone than in an environment without a cell phone.

In this study, individual differences in attentional control function were not assessed by measuring participants’ working memory capacity. Instead, this study focused on how the presence or absence of a cell phone affects attentional control.

## Method

### Participants

For proper counterbalancing, thirty-six adults were recruited through a participant recruitment system provided by the University (*M* = 22.84, *SD* = 2.93, 16 males, 20 females). All participants reported at least 20/40 vision. Additionally, if participants were unable to identify Navon letters (especially small numbers) after completing the practice session, they were informed that they did not qualify as subjects and were not included in subsequent experiments. Participants were compensated 1,000 yen per hour.

### Apparatus

The LCD display (iiyama, Prolite XU2294HS; 499 [W] × 370 mm [H]) was located approximately 60 cm away from the participant’s seat to present the Navon letter. Responses were recorded using a computer-connected keyboard. In the phone-present condition, a cell phone (Apple iPhone 11) was put on the desk and left behind the keyboard. In the phone-absent condition, the phone was replaced by a mobile battery with a size and weight almost identical to that of the phone. Consequently, participants had visual access to the battery or the phone, but they were not allowed to physically touch either of them. The computer-controlled software PsychoPy (ver. 1.90.3) [30, 31] was used to present stimuli on the display and manage the experimental conditions. The Mouse computer (MB-B505S-M2S1) was placed near the display but separated by the partition.

### Stimulus

Global letter/stimulus (3.71° × 2.52°) was consisted of local stimuli (0.41° × 0.32°) and always presented at the central area of the display. In total, the Navon letters have eight patterns: G2/L4 (G: global, L: local), G3/L4, G2/L5, G3/L5, G4/L2, G4/L3, G5/L2, G5/L3. The background color was black.

**Fig. 1:**
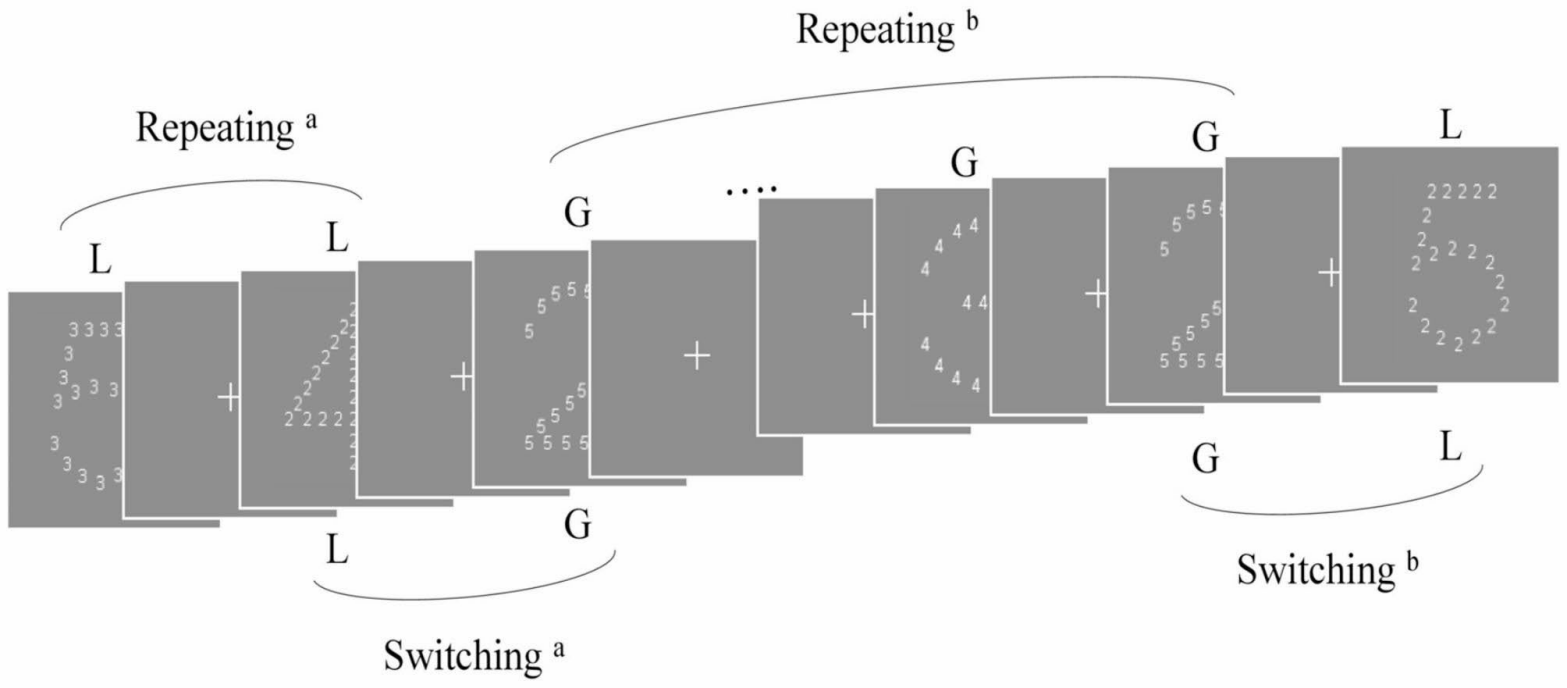
The trial flow of the Navon task. (A fixation point was presented for 1000 ms between each Navon letter presentation. Repeating^a^: the target appeared consecutively in the local level twice, Repeating^b^: the target appeared consecutively in the global level four times; Switching^a^: local-global switching, Switching^b^: global-local switching.)

### Procedure

After completing the necessary participating information, participants took a seat in front of the display, and their heads were secured with a stationary chinrest. After receiving instructions, participants proceeded to a practice session (102 trials, of which 24 were switching trials) and then began the main task. In the main task session, the participants underwent two experimental conditions, with their order counterbalanced among participants. Both conditions consisted of (a) a search-on-phone task where participants were required to use the designated smartphone and (b) a three-block Navon task where participants were not required to use the phone. The sole distinction between the two experimental conditions was whether the cell phone remained on the desk after the search-on-phone task was completed (phone-present condition) or whether the experimenter took it and placed the mobile battery in the same location (phone-absent condition). Throughout the three-block Navon task, the location of the cell phone or mobile battery’s presence remained constant, and participants were instructed not to touch either the cell phone or the mobile battery.

The search-on-phone task (conducted before the Navon task) which was employed in the related study [32] as a dummy task to bring participants’ attention to the cell phone presence. Participants were given a list of 10 Chinese characters and instructed to use the assigned phone to search for 10 commonly used two-word phrases (e.g., “内庭”: inside court) that included the given characters (e.g., “内”: inside). They were given 5 min to collect the phrases and write them in the answer column.

In a trial of the Navon task, a fixation cross was presented for 1,000 ms, followed by the Navon letter for 100 ms. No visual stimuli were presented on the screen until the participant made a response. Participants were instructed to identify which of the two target letters (2 or 3) was present in the Navon letter as quickly and accurately as possible. Irrespective of the level (global or local), when the target was a “2,” they were required to press the F key using their left forefinger, while they were told to touch the J key using their right forefinger when the target was a “3.” The mapping of the response key was counterbalanced between participants. The reaction times and accuracies of the pressed keys were recorded from the onset of the Navon letter.

When the target appeared successively at a certain level, these consecutive trials were defined as one set of repeated-level trials. The target appeared consecutively in a single level was set to either two, four, or six times (Fig. 1). This made it difficult for participants to predict the occurrence of a change in the target’s appearance level would occur. The level of the targets in two consecutive sets of repeated-level trials always differed. Except for the first set of repeated-level trials, the first trial in all sets of repeated-level trials was a switching trial and subsequent trials were repeating trials. In the example in Fig. 1, the first trial of four repeated-level trials was the switching trial (i.e., local-global trial; L-G trials), whereas the second to the fourth trial were the repeating trials (i.e., global-global trials; G-G trials). The composition of Navon letters and the procedure of the Navon task were adapted from [33, 34], and the procedure for level-repetition was adapted from [35]. The target identity was varied randomly, but the target level in the switching trials was strictly controlled. In every type of the repeated-level trials, each target appeared six times at each level. Therefore, participants performed three blocks (25 repeated-level trials per block) in each experimental condition. The sequences of the repeated level trials used in each block that met our experimental requirements were selected using Python (ver. 3.11), and these sequences, used in different experimental conditions, were counterbalanced across participants. They took a 30 s rest between blocks, and a 10-minute rest between experimental conditions. The total number of trials in each experimental condition was 300; however, the first set of repeated-level trials was excluded from the analysis. Therefore, 576 trials in total were included in the analysis. The extent of dependence on the cell phone may influence the effect of cell phone presence, hence the participants also finished the Problematic Use of Mobile Phone (PUMP) questionnaire [36] (translated by a native Japanese speaker) and provided demographic information at the end of the experiment. It took 60 min from the start of the experiment to the end.

### Statistical analyses

Trials with reaction times below 200 ms and those above 3000 ms were interpreted as unsuccessful responses, accounting for 2.8% of all trials, were excluded [37, 38]. The reaction time of correct response and accuracy were analyzed using generalized linear mixed models (GLMM). Data was analyzed using the R software (ver. 3.6.0). GLMM were fitted in R studio software (Version 2021.09.3 + 396) using the function “glmer” in the package “lmerTest” (Version 3.1–2) [39]. Since reaction time distributions are positively skewed [40, 41], a GLMM was modeled for gamma distribution (with an identity link) [42]. The GLMM for accuracy was modeled for binomial distribution (with a logit link). The current study employed the model comparison method (for detailed information, see Results) to determine the main factors influencing participants’ responses. The most appropriate model, which best explains the observed data, was compared using anova(). ANOVA results for the Wald test, using type III sums of squares, were derived from the fitted GLMM models employing the “car” package in R [43]. The multiple-comparison analysis was conducted by using a post-hoc package ‘emmeans’ in R [44]. The p-value was adjusted for multiple comparisons using the Bonferroni correction.

## Results

To test whether the cell phone presence affected the reaction time for correct response and accuracy, the model comparison method was used. The current methodology adheres to a high level of reporting standards [45]. It involves the known factors (trial type, repeated level), the exploratory factors (phone condition, PUMP), and the exploratory interactions (trial type × phone condition, phone condition × PUMP) into the model step by step and selecting the most suitable model to explain the observed data. It is worth noting that while this study does not primarily focus on the effects of cell phone dependency, research on the impact of cell phone presence generally concurs that such effects can occur [10]. Therefore, we included cell phone dependency as an exploratory factor. The model comparison was conducted as follows: Model1 with intercept only; Model2 with trial type (GG, LL, GL, LG; M2); Model3 with trial type and repeated level (2, 4, 6); Model4 with trial type, repeated level, and phone condition (phone-present, phone-absent); Model5 with trial type, repeated level, phone condition, and PUMP score (high, low); Model6 with trial type, phone condition, their interaction, repeated level, and PUMP score; Model7 with trial type, repeated level, phone condition, PUMP score, and their interaction. These models with participants’ ID as a random factor.

As the results of the Wald test using type III sums of squares on the reaction time indicated, Model4 best fits the data of reaction time (Table 1). For Model4, the main effects of trial type, repeated level, and phone condition were significant (trial type: χ^2^ = 748.50, *df* = 3, *p* < 0.001; repeated level: χ^2^ = 8.00, *df* = 2, *p* = 0.018; phone condition: χ^2^ = 16.88, *df* = 1, *p* < 0.001). Multiple-comparison analysis showed that the reaction time increased in the order of trials GG, LL, LG, and GL, irrespective of phone presence (GG vs. GL: 95% CI [− 0.53, − 0.45], z = 24.68, *p*.adj < 0.001, *d* = 0.49; GG vs. LG: 95% CI [− 0.36, − 0.29], z = 16.71, *p*.adj < 0.001, *d* = 0.33; GG vs. LL: 95% CI [− 0.24, − 0.18], z = 15.82, *p*.adj < 0.001, *d* = 0.21; GL vs. LG: 95% CI [0.12, 0.21], z = 6.81, *p*.adj < 0.001, *d* = 0.16; GL vs. LL: 95% CI [0.24, 0.32], z = 14.03, *p*.adj < 0.001, *d* = 0.28; LG vs. LL: 95% CI [0.08, 0.15], z = 5.90, *p*.adj < 0.001, *d* = 0.12). Regardless of trial type, the average reaction time during the two repeated level and four repeated level conditions is longer than during the six repeated level condition (R2 vs. R4: 95% CI [− 0.01, 0.06], z = 1.47, *p*.adj = 0.428, *d* = 0.03; R2 vs. R6: 95% CI [0.01, 0.08], z = 2.73, *p*.adj = 0.019, *d* = 0.05; R4 vs. R6: 95% CI [− 0.00, 0.05], z = 1.58, *p*.adj = 0.344, *d* = 0.02). Additionally, the reaction times in the phone-present condition were faster than those in the phone-absent condition (95% CI [0.02, 0.07], z = 4.10, *p*.adj < 0.001, *d* = 0.05).

**Table 1:** Model comparison analysis (Upper: reaction time, Lower: accuracy)

Model	Fixed effects	AIC	χ^2^	*df*	*p*	
Reaction Time for correct response						
Model1	~ 1	−31,602				
Model2	TT	−32,399	802.89	3	< 2.2e–16	***
Model3	TT + RL	−32,403	7.95	2	0.019	*
Model4	TT + RL + Condition	−32,418	16.88	1	< 3.99e–05	***
Model5	TT + RL + Condition + PUMP	−32,416	0.47	1	0.493	
Model6	TT + RL + Condition + PUMP + TT×Condition	−32,411	0.40	2	0.820	
Model7	TT + RL + Condition + Condition ×PUMP	−32,414	0.09	1	0.759	
Accuracy						
Model1	~ 1	5570.3				
Model2	TT	5398.4	117.91	3	< 2e–16	***
Model3	TT + RL	5401.3	1.08	2	0.582	
Model4	TT + RL + Condition	5401.5	1.82	1	0.177	
Model5	TT + RL + Condition + PUMP	5398.3	5.16	1	0.023	*
Model6	TT + RL + Condition + PUMP + TT×Condition	5400.9	3.08	2	0.214	
Model7	TT + RL + Condition + Condition × PUMP	5400.0	0.35	1	0.552	

TT = trial type; RL = repeated level; Condition = phone condition; PUMP = problematic use of mobile phone; AIC = Akaike information criterion

As indicated by the results of the Wald test using type III sums of squares on accuracy, the model Model5 best fit the data in terms of accuracy (Table 1). Regarding the model Model5, the main effects of trial type and PUMP score were significant (Trial Type: χ^2^ = 182.35, *df* = 3, *p* < 0.001; PUMP: χ^2^ = 5.51, *df* = 1, *p* = 0.019). Multiple-comparison analysis showed that, in both phone condition, the accuracy for the GG trials was higher than the LL, LG, and GL trials (GG vs. GL: 95% CI [1.35, 1.83], z = 13.18, *p*.adj < 0.001, *d* = 1.59; GG vs. LG: 95% CI [0.22, 0.82], z = 3.35, *p*.adj = 0.004, *d* = 0.52; GG vs. LL: 95% CI [0.56, 0.98], z = 7.17, *p*.adj < 0.001, *d* = 0.77), while the accuracy for LL and LG trials was higher than GL trials (GL vs. LG: 95% CI [− 1.36, − 0.78], z = 7.29, *p*.adj < 0.001, *d* = 1.07; GL vs. LL: 95% CI [− 1.02, − 0.62], z = 7.97, *p*.adj < 0.001, *d* = 0.82; LG vs. LL: 95% CI [− 0.02, 0.53], z = 1.80, *p*.adj = 0.426, *d* = 0.26). Furthermore, the accuracy of participants with high PUMP scores was lower than that of participants with low scores, irrespective of phone-present condition (95% CI [− 1.08, − 0.10], z = 2.35, *p*.adj < 0.05, *d* = 0.59).

Taken together, the significantly shorter reaction times in the phone-present condition than in the phone-absent condition and the non-significant interaction between trial type and phone condition indicate that the cell phone presence generally has a facilitating effect on Navon task performance, regardless of the target level (global or local) or inter-trial factors (whether the target level differs from the previous trial). However, neither the reaction time results nor the accuracy results support our hypothesis that the ability to shift visual attention may be facilitated. Interestingly, the results suggest that PUMP did not independently contribute to the change in the reaction time or moderate the influence of the phone condition on reaction time; however, accuracy decreased significantly when the participants’ PUMP score was high. This indicated that cell phone dependence had a negative effect on participants’ responses, but this effect was independent of the impact of the cell phone presence.

## Discussion

This study aimed to investigate whether the cell phone presence can affect the ability to control attentional shifts [15]. Our results indicated that the participants with the phone nearby responded faster when assured that their responses were correct, than they with the mobile-battery nearby. Moreover, this effect was independent of the target level (global or local) and inter-trial factors (whether the target level differed from the prior trial), suggesting that the tendency to respond faster was not limited to switching trials (i.e., GL trials and LG trials), but rather was present in all trial types. Our hypothesis that the cell phone presence may facilitate the ability to shift visual attention, was not supported.

Ward and colleague [6] has suggested that the cell phone presence can reduce working memory capacity, hence this study assumed that the participants in a high working memory capacity state (i.e., phone-absent condition) would not experience a greater attentional shift cost than those in a low working memory capacity state (i.e., phone-present condition). However, the current result is inconsistent with the findings of [25], which shows that high working memory capacity participants experienced greater attentional shift cost than low working memory capacity participants. This discrepancy may be due to the difference in the manipulation of the minority switching trials. In contrast to the random order used in [25], the switching trials in this study always occur after 2, 4, or 6 repetitions. As a result, participants are able to infer that the interval between two target level changes will not exceed a certation duration (approximately 6,000 ms). Alternatively, participants in Goodhew’s study could not predict that switching would occur, whereas participants in this study could predict that switching would occur. Goodhew suggested that high working memory capacity individuals might allocate attentional resources to facilitating target levels to improve their performance on the majority trials, resulting in low performance on the minority switching trials. In this case, however, individuals would not prefer a strategy that allocates attentional resources to a particular target level because it could not help improving their performance when the target level changes in a routine. Thus, Goodhew’s findings highlight the effect of a reduction in resources that can eliminate the attentional cost that arises from a random shift, which is absent in the current case. Accordingly, we cannot rule out the possibility that the difference in manipulation leads to a null effect on the ability to shift visual attention, which is a limitation of the current study. Further research should investigate cases in which the attentional shifts are caused by an accidental target level switching.

Although the attentional shift hypothesis was not supported, the current findings still extended previous findings on the influence of the cell phone presence on visual attention by finding that the cell phone presence can facilitate the perceptual process of a stimulus when it appears only in the central visual field. This was inconsistent with previous studies that reported a negative effect of cell phone presence on visual search, which also relies on visual attention [4]. These inconsistent findings may be due to the fact that the visual search may be affected not only by the efficiency of the perceptual process of stimulus but also by the number of perceptible stimuli, which is limited by the attentional scope. In a visual search task, participants typically direct their attention to a stimulus-centered area (i.e., attentional scope) and identify whether the stimulus in that area is the target stimulus. If the target is not found in the current area, it is necessary to shift attention to another stimulus-centered area (i.e., eye movement) and identify the stimulus again in the new stimulus-centered area again (re-perceptual process). In addition to the dramatic reduction in visual search times facilitated by other factors (e.g., the random situation in which a target stimulus is located right within the initial stimulus-centered area), the reduced attentional scope implies that the number of stimuli that can be perceptually processed at one time is reduced, and the times for re-perceptual processes subsequently increase. Given that the influence of the cell phone presence is more likely to manifest its effect on attentional resources which are distributed in the peripheral visual field [46, 47], the attentional scope decreases when the phone is present. Thus, it is possible to believe that the effect of cell phone presence on the attentional scope may increase the re-perceptual processing times, resulting in a longer visual search time, which may be a more plausible explanation for the effect of cell phone presence on the visual search task. Moreover, it is notable that the effect of cell phone presence reported in Ito and Kawahara’s study (approximately 1,000 ms) is much larger than the facilitation effect on perceptual processing in the current case (estimated marginal means: 12 ms). Thus, the facilitating effect may be relatively small, making it difficult to detect in the case of a visual search task.

Instead, the current results showed two unexpected effects associated with the cell phone presence. First, the cell phone presence is associated with a general facilitatory effect on the perceptual-motor performance in the Navon task. Typically, anxiety triggered by participants’ fear of missing a message on their cell phone [48] is often considered as the reason for quicker reaction times. However, this is not applicable in our case because the phones used in the current study were not owned by the participants. Consequently, even with the presence of cell phones, it was challenging to cause concern among the participants, and it is hardly conceivable that a decrease in reaction time in the phone-present condition was due to anxiety. Alternatively, another possible explanation can be suggested. Visual attentional resources are mainly distributed in the central area of vision, with the resources that are allocated in the peripheral visual field decreasing with distance from fixation. When available attentional resources are decreased, the cognitive system may prioritize reducing the amount of attentional resources distributed in the peripheral visual field to maintain the perceptual processing of information in the central area of vision [49]. In one related study [46] on the impact of the cell phone presence on the allocation of attention in the visual field, it was found that only the attentional resources allocated in the peripheral visual field are significantly reduced by the cell phone presence, while the attentional resources allocated in the central area of vision are slightly increased. It suggested the effect of cell phone presence on the distribution of attentional resources, i.e., participants can dramatically adjust their attentional resources to the central area of vision to avoid the distraction of the cell phone presence, which may lead to an improvement in task performance for the central visual field. In the current study, the Navon letter was always present in the central area of vision; therefore, a possible explanation for the current results is that the cell phone presence caused the attentional resources to converge on the fixation, and the reaction time for both types of trials became fast because more resources were available for perceptual processing. This result also suggests that the effect of cell phone presence should be a mixed one. In other words, in certain specific cases, the presence of a cell phone may have both negative and positive effects simultaneously, resulting in no significant change in cognitive task performance. Therefore, the discovery of a facilitating effect can help elucidate the findings of previous studies [50, 51] that failed to observe the impact of cell phone presence in a more comprehensive manner.

Second, this study found that participants with high dependence were less accurate on tasks, suggesting that dependence on cell phones affects tasks that require attentional control. However, since this negative effect was found independent of the cell phone presence, it can be considered to be a long-term effect of cell phone use habits rather than a direct effect of cell phone presence. In this study, the designated cell phone was not personally owned by the participants, which may have potentially influenced the results. If they had owned the phones they were using, the bond between the cell phones and the participants would be stronger. This could lead to the cell phone presence increasing the effects of cell phone dependence. Therefore, future research investigating the effect of cell phone dependence on cognitive performance should consider phone ownership as a potential moderator.

This study has several limitations that should be noted. First, although the cell phone dependency scale used in this study is reliable, as concerns regarding cell phone usage increase, a recent systematic review of problematic cell phone usage scales has pointed out the lack of sufficient internal consistency and test-retest reliability in existing self-reported cell phone dependency scales during actual use [52]. Studies focusing on the impact of cell phone dependence should fully consider this issue. Second, simultaneous use of attention control tasks and working memory capacity tasks can help further explain the differences in the effects of cell phone presence on various cognitive activities. Additionally, while this study briefly introduced the relationship between cell phone presence, attention, and cognitive performance, it is actually far more complex than that. Future research needs to pay closer attention to explaining these relationships.

## Conclusion

Given that the attentional cost of the cell phone presence may negatively affect the efficiency of cognitive activities, some scholars have suggested ensuring restrictions on their presence in the work environment. This study demonstrates the possibility of refuting the claim that cell phones must always be excluded from the workplace by highlighting the positive effects of the cell phone presence. Cell phones affect our cognitive activities, and it is sometimes useful to remove cell phones to reduce the disruptive effects. However, it is natural for workers to want to have their cell phones nearby. Therefore, future investigations are required to better understand the effects of cell phones and to find optimal usage.

## Data Availability

The datasets supporting the conclusions of this article are available in the Open Science Framework, https://osf.io/9bhgw/?view_only=9a87a79acfdc46d1a4f1408acc2b227b.
